# Seeing Your Foot Move Changes Muscle Proprioceptive Feedback

**DOI:** 10.1523/ENEURO.0341-18.2019

**Published:** 2019-03-22

**Authors:** Rochelle Ackerley, Marie Chancel, Jean-Marc Aimonetti, Edith Ribot-Ciscar, Anne Kavounoudias

**Affiliations:** 1 Aix-Marseille Université, Centre National de la Recherche Scientifique, Laboratoire de Neurosciences Sensorielles et Cognitives - UMR 7260, Marseille 13331, France; 2 Department of Physiology, University of Gothenburg, Göteborg 40530, Sweden; 3 Department of Neuroscience, Karolinska Institutet, Stockholm 17177, Sweden

**Keywords:** fusimotor drive, human, kinesthesia, movement perception, muscle proprioception

## Abstract

Multisensory effects are found when the input from single senses combines, and this has been well researched in the brain. Presently, we examined in humans the potential impact of visuo-proprioceptive interactions at the peripheral level, using microneurography, and compared it with a similar behavioral task. We used a paradigm where participants had either proprioceptive information only (no vision) or combined visual and proprioceptive signals (vision). We moved the foot to measure changes in the sensitivity of single muscle afferents, which can be altered by the descending fusimotor drive. Visual information interacted with proprioceptive information, where we found that for the same passive movement, the response of muscle afferents increased when the proprioceptive channel was the only source of information, as compared with when visual cues were added, regardless of the attentional level. Behaviorally, when participants looked at their foot moving, they more accurately judged differences between movement amplitudes, than in the absence of visual cues. These results impact our understanding of multisensory interactions throughout the nervous system, where the information from different senses can modify the sensitivity of peripheral receptors. This has clinical implications, where future strategies may modulate such visual signals during sensorimotor rehabilitation.

## Significance Statement

It is well known that multisensory processes occur in the brain, yet we know little about the consequences of multisensory interactions at the spinal level. We recorded from single muscle afferents, while participants either saw or did not see their foot moving. We show that adding visual information reduces muscle afferent firing, probably via descending commands by fusimotor efference. These results impact sensorimotor rehabilitation, where clinical strategies using exercises without visual feedback may promote proprioceptive training.

## Introduction

Perception is multimodal by nature and the CNS integrates multiple sensory sources to produce coherent percepts (Kavounoudias, 2017). Combining spatially and temporally congruent multisensory cues is beneficial (Stein and Stanford, 2008), where combined vision and muscle proprioception can improve perceptual or motor responses (Tardy-Gervet et al., 1986; Rossetti et al., 1995; Van Beers et al., 1999; Sober and Sabes, 2003; Reuschel et al., 2010; Guerraz et al., 2012; Blanchard et al., 2013). These studies have shown that convergent inputs must be integrated properly to assess body configuration and any changes that may occur. Computational modeling, in particular the theoretical Bayesian framework, provides such an approach to predict perceptual enhancement due to multisensory integration, by postulating that the multisensory estimate of an event is given by the reliability-weighted average of each single-cue estimate (Ernst and Banks, 2002; Landy et al., 2011). Bayesian predictions have shown the optimal integration of vision and proprioception when evaluating arm movements (Reuschel et al., 2010), positions in space (Van Beers et al., 2002; Holmes and Spence, 2005; Tagliabue and McIntyre, 2013), and in performing pointing motor tasks (Sober and Sabes, 2003).

Interactions between sensory systems are found in the brain, including in the early stages of sensory information processing (Kavounoudias et al., 2008; Cappe et al., 2009; Hagura et al., 2009; Helbig et al., 2012; Klemen and Chambers, 2012). The sensitivity of muscle afferents can be modulated via central efference, which may mean that the periphery is subject to multisensory influences. The fusimotor system sends efferent γ-motoneurons from the spinal cord to the intrafusal fibers of muscle spindles (Awiszus and Schäfer, 1989; Murphy and Martin, 1993; Ellaway et al., 2002, 2015), where the positional sensitivity of muscle afferents is changed by γ-static fusimotor neurons and their velocity sensitivity by γ-dynamic fusimotor neurons (Matthews, 1981).

Since direct recordings of γ efferents are rare in humans (Ribot et al., 1986), the influence of the fusimotor drive is classically assessed by recording the activity of single muscle afferents, whose modulation can likely be indirectly supported by a change in the fusimotor drive. Through this approach, microneurographic studies have shown that the fusimotor drive can influence muscle afferent firing depending on the attentional (Hospod et al., 2007; Ribot-Ciscar et al., 2009) or emotional (Ackerley et al., 2017) context. Hospod et al. (2007) showed a decrease in the dynamic sensitivity of primary muscle afferents when a participant’s attention was selectively directed to the recognition of an imposed, complex, two-dimensional movement. Conversely, muscle afferent dynamic sensitivity has been observed to increase when the proprioceptive attention task was specifically oriented towards the movement velocity (Ribot-Ciscar et al., 2009). These studies show an independent static or dynamic fusimotor control of muscle spindle sensitivity in humans, which depends on the behavioral context.

There are few studies on the influence of vision on muscle proprioceptive sensitivity via the fusimotor drive. Wessberg and Vallbo (1995) compared muscle afferent activity from the hand during a visual tracking task that consisted of following a target displayed on a screen; during the reproduction of the same movement in the absence of visual control, no difference was reported. In contrast, Jones et al. (2001) showed that muscle afferent activity decreased in a visuo-motor adaptation task, where the displacement of a visual target was shifted, making the visual information incongruent with proprioceptive information from the moving hand. The decrease in proprioceptive sensitivity was interpreted as a strategy for resolving bisensory conflict. More recently, Dimitriou (2016) showed that the muscle spindle firing varied with adaptation state independently of muscle activity, making the γ system a specific contributor to motor learning.

In these previous studies, vision was not directed towards the participant’s own moving body, but towards a visual target (displaced by the participant’s moving hand). In addition, these studies used active, rather than passive movements. Active movements are more representative of natural body conditions; however, passive movements are ideal to address muscle spindle sensitivity in the absence of concomitant activation of skeletomotor neurons (α-γ coactivation). Presently, we investigated whether seeing your own foot move passively altered muscle proprioceptive feedback and how it might be related to perceptual performance. We designed a behavioral experiment to test whether movement amplitude discrimination was better when participants viewed their foot moving, as compared to only having muscle proprioception when participants kept their eyes closed. Further, we examined changes in muscle spindle sensitivity to similar passively-imposed foot movements, varying both vision and attention, where we hypothesized that muscle afferent firing would be modulated over these conditions.

## Materials and Methods

The present experiments were performed on healthy human volunteers [human subjects were recruted at Aix-Marseille University], where written, informed consent was obtained and a random experimental design was used. The study was approved by the local ethics committee [Comité de protection des personnes Sud-Méditerranée I, Marseilles] and performed in accordance with the Declaration of Helsinki. The study consisted of two series of experiments to investigate the multisensory effects of visual and proprioceptive processing: one using behavioral psychophysics and the other using the *in vivo* technique of microneurography. Fifteen volunteers (two males; 26 years ± 5 SD) took part in the first behavioral experiment, and 13 (seven males; 26 years ± 6 SD) different volunteers took part in the second microneurographic experiment.

### General experimental set-up

In both experiments, the participants were seated in a semi-reclined armchair with their legs positioned in cushioned grooves, so that a standardized position could be maintained without muscle activity. The knee joint was at a flexion angle of ∼120–130°. The right foot rested on a stationary plate and the left foot rested and was held on a pedal connected to a computer-controlled robot, allowing sinusoidal foot plantar flexion/dorsiflexion movements to be imposed. The absence of concomitant muscle activity was monitored throughout the two experiments by recording surface electromyography (EMG). A pair of surface electrodes (Ag–AgCl, interelectrode distance 2 cm) was placed over the tibialis anterior (TA) and another pair on gastrocnemius soleus (GS) muscle bellies during the behavioral experiment. In the microneurographic experiment, pairs of surface electrodes were placed over the TA [corresponding to afferents originating in TA and extensor digitorum longus (EDL) muscles] and peroneus longus (PL; corresponding to afferents originating in PL) muscle bellies. The location of each pair of electrodes was defined by asking the participant to isometrically contract the muscle under consideration and palpation of the muscle belly. The EMGs were band-pass filtered (30–3000 Hz), recorded with a high gain (5000×), and sampled at 10 kHz. Autonomic responses were recorded through electrodermal activity (EDA), using two surface electrodes placed on each side of the left hand (gain: 500×, band-pass: 0.1–100 Hz, sampling frequency: 500 Hz). Physiological data were stored on a digital tape recorder (DTR 1802, Biologic) and processed off-line in Spike2 (Spike2 Software, RRID:SCR_000903). During all experiments, participants wore noise-cancelling headphones (Bose) to prevent extraneous sounds.

### Unitary muscle afferent recordings

The *in vivo* technique of microneurography was used to record from the left common peroneal nerve at the popliteal fossa in humans (Hagbarth and Vallbo, 1968; Bergenheim et al., 1999). The nerve was located by palpation. Unitary muscle afferent activity was recorded differentially using an insulated tungsten microelectrode (impedance 0.3–1 MΩ, tip diameter ∼5 µm, length ∼30 mm; FHC). The recordings were monitored continuously using an oscilloscope and a loudspeaker. Neural activity was amplified (100,000×) and band-pass filtered (300–3000 Hz) to ensure an optimal signal-to-noise ratio and sampled at 20 kHz. Muscle afferents were identified as primary endings on the basis of their irregular spontaneous activity, their high dynamic sensitivity to ramp-and-hold movements, and their silencing during passive muscle shortenings (Edin and Vallbo, 1990). The activity from 24 single muscle spindle endings (21 Type Ia muscle afferents and three Type II) was recorded, but due to a loss of unit stability over time in some recordings, we gained full datasets over all conditions (vision, no vision, attention, no-attention) from 16 units (all Type Ia). These originated in the EDL (*n* = 10), PL (*n* = 3), and TA (*n* = 3) muscles. Microneurographic data were stored via digital tape recorder (DTR 1802, Biologic), along with the physiological data. Data were processed off-line by means of Spike2 Software (RRID:SCR_000903).

### Procedure

#### Behavioral experiment

Participants were required to discriminate the amplitude difference between two imposed movements of their left foot. The robot moved their foot up-and-down twice, which then returned to its initial position (set at 20° and 40° from typical maximal dorsal and plantar flexions, respectively). The velocity was fixed at 5°/s. One of the movements was always the same reference movement, corresponding to an amplitude angle of 6.4° between the foot and the shin bone. Before each movement pair (repeated 15 times), participants were orally instructed to keep their eyes closed (“no vision,” proprioceptive-only information) or have them open (“vision,” combined and congruent visuo-proprioceptive information); vision and no vision trials were randomized. In the vision condition, the participants were required to look at their left foot moving. Each trial included the reference movement at 6.4° (given randomly the first or second movement) and another “test” movement, which consisted of one of seven possible angles (5.1°, 5.6°, 6°, 6.4°, 6.8°, 7.2°, or 7.6°). These angles were chosen on the basis of a previously defined pilot study (performed on four participants not included in the main experiment), in order to identify angle amplitudes that make discrimination against the 6.4° reference very difficult (6° and 6.8°) or rather easy (5.1° and 7.6°) or of intermediate difficulty (5.6° and 7.2°). Participants had to decide whether the first or the second movement was the largest in amplitude. They answered orally “one” or “two,” after the movements had finished, when prompted by the experimenter. Each angle was tested 30 times (15 times with closed eyes and 15 times with opened eyes) and resulted in a total of 210 movement comparisons (30 repetitions × seven angles) per participant. All movement pairs were pseudo randomized. Three-minute breaks were systematically given after every 20 pairs of movement comparisons and the experimenter regularly checked whether the subject needed to take an extra break at any time to prevent fatigue and loss of motivation.

#### Microneurographic experiment

Participants underwent similar passive foot displacements at the level of the ankle, where a series of 30 sinusoidal plantar flexion/dorsiflexion movements (5° amplitude and 5°/s velocity, over ∼1 min) were imposed during microneurographic recording. This longer foot movement protocol was chosen for the single unit microneurographic recording because it was important to analyze muscle afferent firing in the absence of muscle activity. A time pause of 30 s was given after each movement.

To investigate the effect of vision, the activity of each muscle afferent was recorded under four conditions presented in a pseudo-randomized order using a 2 × 2 factorial design, with vision (vision, no vision) and attention (attention, no attention) as experimental factors. Visual information was manipulated by asking the participant either to keep their eyes closed (no vision condition), or their eyes open with the instruction to watch the movement of their foot (vision condition). Attention was manipulated by asking the participants either to simply relax and not pay attention to their foot moving (no attention condition) or they were instructed to pay attention to the movement of their foot (attention condition). To make sure that the participants were attentive, the participant was asked to judge whether it felt like the current sinusoidal movements were of larger amplitude than the previous ones. In fact, it was always the same passive movement imposed on the participant, to compare the response of muscle afferents to investigate a change in firing properties of the afferent fibers depending on the experimental conditions. Therefore, the same movement amplitude was used over all the four experimental conditions in the microneurographic study. We chose the lowest amplitude (5°) from the range of amplitudes previously tested in the present psychophysical study. Only one amplitude was used to minimize the duration of the experiment, as the longer the microneurographic recording, the higher the risk of losing the unit (e.g., due to electrode displacement) and thus not obtaining data. This is a common risk during microneurography in humans, which was more likely to occur presently due to the long-lasting trials used in this study (30 cycles of 189 ankle movements, repeated). In addition, to avoid any implicit attention task, the no-attention and attention trials were blocked separately, and the no-attention block always preceded the attention block.

### Data analysis

Data were analyzed in MATLAB (RRID:SCR_001622) and compared statistically in SPSS (RRID:SCR_002865) with a level of significance set at *p* < 0.05. For all statistical tests, effect sizes were determined using partial η^2^. See the statistical table (Table 1) for further details of the tests carried out.

**Table 1.** Data structure for statistical analyses

	Data structure	Type of test	Power
a	Behavioral amplitude discrimination level (*n* = 15 participants)	GzLM	0.5
b	EMG and EDA data tests per condition/variable (*n* = 15 participants) for behavioral experiment	Student’s paired *t* test	0.5
c	Microneurography data for change in muscle afferent firing over conditions (*n* = 16 units)	Repeated measures two-way ANOVA	0.6
d	EMG and EDA data tests per variable (*n* = 16 recordings) for microneurography experiment	Repeated measures two-way ANOVA	0.6

Type and power of the statistical tests carried out in the psychophysics and microneurography experiments. Letters in the left column refer to values within the Results section.

#### Behavioral experiment

In order to evaluate and compare participants’ performances across the two conditions (vision/no vision), we used an approach classically employed to estimate velocity discriminative thresholds of self-movements (Wichmann and Hill, 2001; Ernst and Banks, 2002; Kingdom and Prins, 2009; Reuschel et al., 2010; Tagliabue and McIntyre, 2013; Chancel et al., 2016a; Landelle et al., 2018). The psychometric data (i.e., the proportion of answers corresponding to movements found to be larger in amplitude than the reference) were fitted by a cumulative Gaussian function:

$$P\left(x\right)=\lambda +\left(1-2\lambda \right)\frac{1}{\sigma_{\Psi }\sqrt{2\pi }}\int_{-\infty }^{x}e^{-\frac{{\left(y-{µ}_{\Psi }\right)}^{2}}{2\sigma_{\Psi }^{2}}}dy$$

Here, *x* represents the movement angle (in degrees); μ_ψ_ is the mean of the Gaussian, i.e., the point of subjective equality (PSE), that corresponds to the stimulation intensity leading the participant to perceive no difference between the reference and the test movements; and σ_ψ_ is the standard deviation (SD) of the curve (discrimination threshold), which is inversely related to the participant’s discrimination sensitivity. A smaller σ_ψ_ value corresponds to higher discrimination sensitivity in the task and was used to measure their discrimination capability. The two indices, PSE and σ_ψ_, characterize the participant’s performance, and λ accounts for stimulus-independent errors (e.g., due to participant lapses) and was restricted to small values (0 < λ < 0.06; Wichmann and Hill, 2001). This parameter is not informative about the perceptual decision, thus we disregarded it for the subsequent analyses. Psignifit toolbox, implemented in MATLAB, was used to fit the psychometric curves. In this fitting procedure, bootstrap analysis was performed and the goodness-of-fit of the chosen model (i.e., the Gaussian function) was checked. As a result, the statistical power of the two parameters obtained to describe each participant’s perception, mean and variance, was reinforced which leads to a reliable comparison between the different conditions both within and between participants (Wichmann and Hill, 2001). Since the σ_ψ_ (β) values can be assimilated as positively-skewed continuous variables modeled by a γ distribution, we used a non-parametric generalized linear model for repeated measured (GzLM) to compare these variables between the vision and no vision conditions.

#### Microneurographic experiment

The nerve spikes were inspected carefully for their single unit nature in an expanded time scale and then transformed into an instantaneous frequency curve (bin size = 0.005 s). The mean curve was obtained by averaging the response to 29 sinusoidal movements, where the first movement was excluded because of a dynamic response from the onset of the movement. Occasionally, some EMG activity (i.e., fluctuations in the steady EMG baseline) was found, despite the instruction for the participant to relax. When this occurred, the contaminated movement cycle was removed (Ackerley et al., 2017). This occurred in only 5/64 runs (16 units × four conditions) and for each case, at least 85% of cycles were included. Measures were extracted from the averaged response, including the maximum and minimum frequency, and the difference between these two measures (“δ”), which was used as an index to characterize a unit’s response in each condition (Ackerley et al., 2017). This measure was used to quantify the dynamic response of muscle afferents (Kakuda, 2000).

In line with other microneurographic studies of muscle afferent firing (Dimitriou, 2016), the data were normalized (z-transformed to give z-scores), so as to compare differences across the conditions over the individual afferents. Here, we obtained the δ per afferent/condition, which was then normalized by subtracting the mean δ, and this was divided by the δ SD, for that afferent. This produced the number of SDs by which each condition differed from the mean value for each afferent tested. Statistical analyses were conducted on these normalized data, on the whole population of afferents, where the data were first checked for normality. A repeated measures two-way ANOVA was carried out in SPSS, to determine the effects of visual information and attention, and any interaction between these.

#### Physiological indexes in both experiments

The EMG and EDA activity were used to investigate whether the participant showed muscular or autonomic activity in the experiments. The direct current offset was removed from the EDA data and the EDA and EMG data were down-sampled to 2.5 kHz. For the psychophysical experiment, these data were separated by visual condition, where data were epoched from the beginning of the movement to the end of a movement, per trial, resulting in 105 total trials for the combined visuo-proprioceptive information condition and 105 for the proprioceptive-only condition. For the microneurographic experiment, EMG (one EMG source was used, which depended on the muscle afferent recorded from) and EDA signals were extracted, per condition per participant, from the duration of the sinusoidal movement. For both signals, areas under the curves were measured to analyze the modulation of physiological signals across conditions. The mean values, per measure, were checked for normality and the visual conditions were compared by Student’s paired *t* tests in the behavioral experiment and the visual/attention conditions using repeated measures two-way ANOVA for the microneurography experiment.

## Results

### Behavioral measurement of effect of visual information on movement discrimination

**Figure 1.**
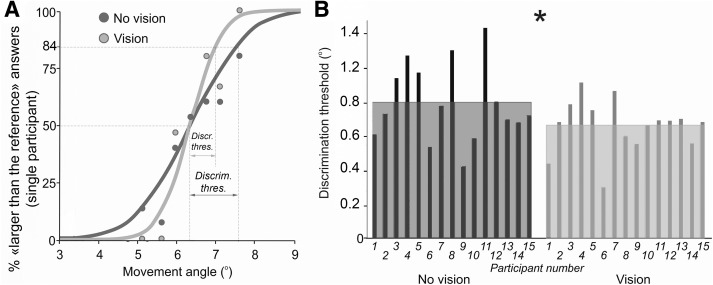
Behavioral effects of visual information on foot movement amplitude discrimination. ***A***, An example of the mean psychometric curves for a single participant, where the slope is significantly steeper (lower amplitude discrimination threshold (Discrim.thres.)) when they saw their foot moving. ***B***, For the group (*n* = 15 participants, shown in individual bars), there was a significant decrease in the discrimination threshold of movement amplitude when the participant watched their foot moving, as compared to having their eyes closed and only using proprioceptive information (*p < 0.05 and the mean discrimination levels are shown as boxes).

Figure 1*A* shows an example of a participant’s ability to discriminate the amplitude of their foot movement. The discrimination improved in the visuo-proprioceptive condition, compared to the proprioception-only condition, as shown by an increased slope of the visuo-proprioceptive psychometric curve. More precisely, the discrimination threshold σ (i.e., the increase in movement amplitude required to induce a perception of movement larger than the reference movement in 84% of the trials with respect to 50% of the trials) was lower in the visuo-proprioceptive condition. The group data revealed that participants were on average able to discriminate the angle of their foot with higher precision in the vision condition, as compared to the no vision condition, as shown by a decrease in the discrimination level (Fig. 1*B*). The discrimination threshold σ was significantly lower in the vision condition (mean σ = 0.66 ± 0.04° SEM (standard error of the mean)) than in the no vision condition (mean σ = 0.8 ± 0.06° SEM; GzLM analysis slope = 0.242, *t* = 3.31 *p* < 0.001; Fig. 1*B*, Table 1, row a). No significant differences were found in the physiological measures (EMG, EDA) between the visual conditions (Table 1, row b, Table 2).

*Figure Contributions:* Marie Chancel performed the experiment. Marie Chancel and Anne Kavounoudias analyzed the data.

**Table 2.** Mean values and statistics for the physiological measures during microneurography experiment

	EMG (mean ± SEM)	EDA (mean ± SEM)
No vision, relax	14,105 ± 2927	45,857 ± 1537
No vision, attention	14,073 ± 2925	45,828 ± 1502
Vision, relax	14,105 ± 2899	45,820 ± 1555
Vision, attention	14,093 ± 2897	45,723 ± 1556
ANOVA main effect vision	*F*_(1,15)_ = 3.45, *p* = 0.081, partial η^2^ = 0.20	*F*_(1,15)_ = 0.31, *p* = 0.568, partial η^2^ = 0.02
ANOVA main effect attention	*F*_(1,15)_ = 0.34, *p* = 0.857, partial η^2^ = 0.01	*F*_(1,15)_ = 0.18, *p* = 0.679, partial η^2^ = 0.01
ANOVA interaction vision × attention	*F*_(1,15)_ = 0.17, *p* = 0.683, partial η^2^ = 0.01	*F*_(1,15)_ = 0.12, *p* = 0.739, partial η^2^ = 0.01

The mean values for the EMG and EDA, with the SEM, as shown for the microneurography experiment. The EMG and electrodermal responses are shown in arbitrary units (area under the curve) for the duration of the sinusoidal cycles per condition. There was no significant effect of vision, attention, or the interaction of these, as shown in the ANOVAs, where the partial η^2^ shows the size effects.

### Microneurography measurement of effect of visual information on movement encoding

**Figure 2.**
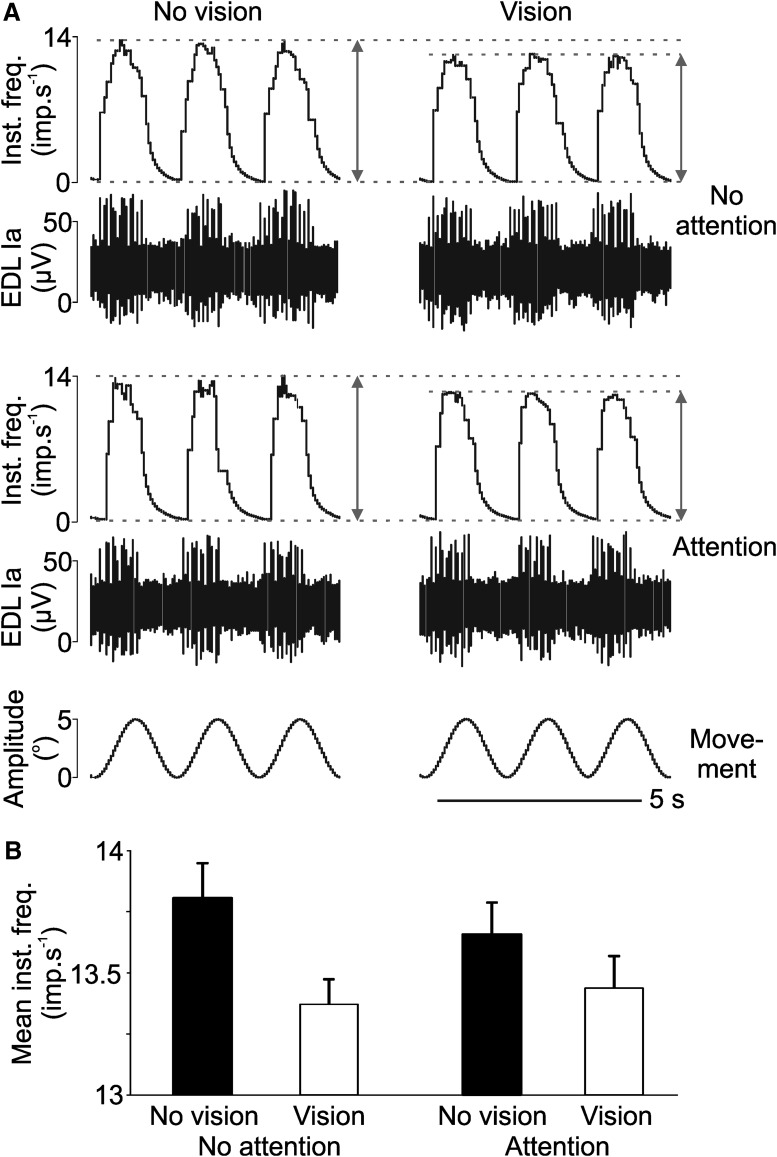
An example of muscle afferent firing, physiological measures, and the differences between conditions in a single participant. ***A***, An example of three consecutive sinusoid movement cycles applied during each of the four visual/attention conditions. The minimum and maximum firing rates were extracted (gray arrows at the end of each example) and this mean firing rate change (δ) was used to quantify the dynamic response of the muscle afferent, for each condition. In this example, a microneurographic recording was made from a primary muscle afferent (Ia) arising from extensor digitorum longus (EDL) muscle. ***B***, For this muscle afferent, a clear difference in the δ can be seen between when the participant had visual or no visual information (Histograms are mean values and bars are standard deviations per condition), regardless of the attention condition. Inst. freq: instantaneous frequency; imp.: impules.

A total of 16 primary Ia muscle afferents were tested over the conditions where the participant viewed their foot moving (vision) or had their eyes closed (no vision), during a further task of paying attention to the movement (attention) or not (no attention). Figure 2 shows examples of unitary recordings from a muscle afferent over the conditions (Fig. 2*A*), with the mean extracted change in instantaneous firing (δ) over the sinusoidal movement cycles per condition (Fig. 2*B*). It can be seen in, both the individual cycles and in the unit’s means, that there was a clear difference between the vision conditions, where the mean instantaneous firing frequency was lower with visual information, in both attention and no attention conditions.

*Figure Contributions:* Rochelle Ackerley, Edith Ribot-Ciscar and Jean-Marc Aimonetti performed the experiments. Rochelle Ackerley, Edith Ribot-Ciscar and Anne Kavounoudias analyzed the data.

**Figure 3.**
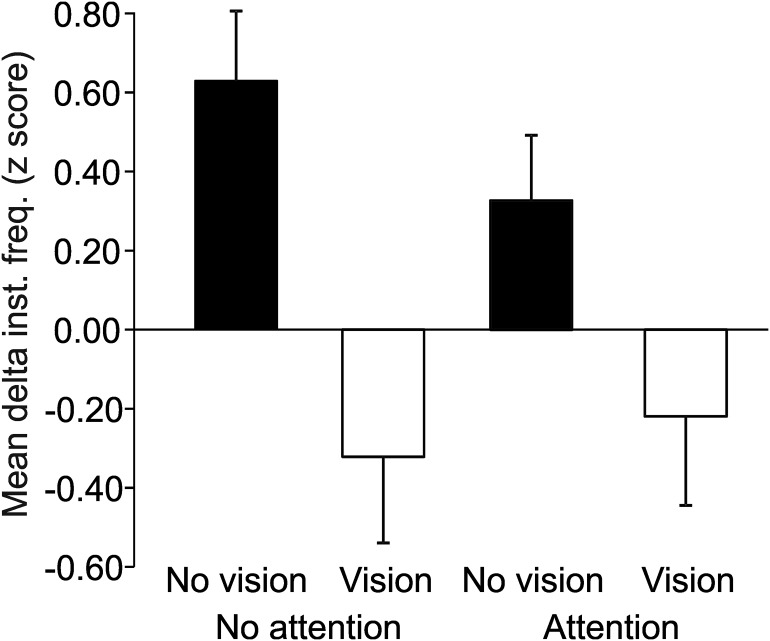
The mean effect of visual information and attention on muscle afferent movement encoding. The group data of Ia muscle afferents (*n* = 16) show a significant difference in the dynamic response of muscle afferents, as measured by the change in the minimum-to-maximum firing rates (δ), which was normalized via z-transform (means and SEMs are shown). A main effect was found for having visual information, where the δ was significantly lower with visual information, but no significant difference was found in the response between attention conditions, nor the interaction between vision and attention. Inst. freq. : instantaneous frequency.

The same result was found in the group data (Fig. 3). A repeated measures ANOVA on the δ z-scores showed a significant main effect of vision (*F*_(1,15)_ = 20.36, *p* < 0.001, partial η^2^ = 0.58; Fig. 3), but no significant effect of attention (*F*_(1,15)_ = 0.19, *p* = 0.672, partial η^2^ = 0.01), nor an interaction between visual information and attention (*F*_(1,15)_ = 0.64, *p* = 0.435, partial η^2^ = 0.04; Table 1, row c). Therefore, a significant increase in δ was found when visual information was removed, but paying attention to the movement did not make a difference in the muscle afferent firing in this paradigm.

*Figure Contributions:* Rochelle Ackerley, Edith Ribot-Ciscar and Jean-Marc Aimonetti performed the experiments. Rochelle Ackerley, Edith Ribot-Ciscar and Anne Kavounoudias analyzed the data.

The physiological data (EMG, EDA) showed no significant differences between the conditions (Table 1, row d, Table 3). Here, for both EMG and EDA data, we found no significant effect of having visual information, or not, and neither was there an effect of whether the participant paid attention to the movement or simply relaxed, nor an interaction of these factors.

**Table 3.** Mean values and statistics for the physiological measures during behavioral experiment

	EMG TA (mean ± SEM)	EMG GS (mean ± SEM)	EDA (mean ± SEM)
Vision	9330 ± 1146	8478 ± 1766	8217 ± 1595
No vision	9237 ± 1076	8343 ± 1076	7827 ± 1237
Paired *t* test vision vs no vision	*t*_(13)_ = 0.92, *p* = 0.385, partial η^2^ = 0.06	*t*_(13)_ = 1.82, *p* = 0.092, partial η^2^ = 0.20	*t*_(13)_ = 0.64, *p* = 0.531, partial η^2^ = 0.03

The mean values for the EMG and EDA, with the SEM, as shown for the behavioral experiment. The EMG and electrodermal responses are shown in arbitrary units (area under the curve) for the total number of trials per condition. There was no significant effect of vision, as shown in the ANOVAs, where the partial η^2^ shows the size effects.

## Discussion

Presently, we investigated the effect of congruent visual and/or proprioceptive signals on the processing of ankle movement. We found that visual information interacted with proprioceptive information, as seen in behavioral measures and in the responses of single muscle afferents. When participants saw their moving foot, they were more accurate in judging movement amplitude. Further, we found that the response from single muscle afferents was increased when the proprioceptive channel was the only source of sensory information, as compared to when the participant had the congruent visual input.

### Enhancement of visuo-proprioceptive perception

Our behavioral results confirmed that combining visuo-proprioceptive information relating to self-body movements provided a perceptual enhancement, as there was a significant decrease in the threshold for discrimination when additional visual information was available. This corresponds well with many studies showing that combining congruent visuo-proprioceptive stimulation enhances the resulting perception, suggesting that both visual and proprioceptive cues are co-processed for kinesthetic purposes (Tardy-Gervet et al., 1986; Rossetti et al., 1995; Van Beers et al., 1999; Reuschel et al., 2010; Guerraz et al., 2012; Blanchard et al., 2013). For example, using the classical mirror paradigm, Guerraz et al. (2012) reported that when participants looked at the reflection of their moving left arm in a mirror, they felt an illusion of a concomitant displacement of their stationary, hidden right arm. When a congruent muscle vibration was added on the resting right arm, i.e., simulating a movement in the same direction as that of the visual moving arm, the velocity of the resulting illusion increased, showing the beneficial impact of multisensory inputs.

Vision and muscle proprioception may combine in movement perception, but it does not mean that the weights allocated to each of these sensory cues are equal. For example, under artificial conflicting visuo-proprioceptive conditions, where visual cues of the participants were deviated using prisms and participants had to place an unseen finger in the same position as their seen finger, visual information has been shown to override muscle proprioceptive information under full-light conditions. In contrast, proprioception dominates when vision input is severely reduced to a small light-emitting diode on the end of their finger, viewed in darkness (Plooy et al., 1998). Therefore, the exact behavioral context must be taken into account. According to the theoretical Bayesian framework, the CNS allocates relative weights to each sensory cue on its relative reliability to encode the perceptual event in a given context and their weighted combination can optimize the resulting perception (Ernst and Banks, 2002; Landy et al., 2011). Although our present experiment was not designed to test the optimality hypothesis, one may hypothesize that the discriminative enhancement we found in the bisensory condition may be explained by a weighted combination of both visual and proprioceptive information, as reported in other perceptual tasks (Van Beers et al., 1999; Reuschel et al., 2010).

### Dynamic muscle spindle sensitivity increases in absence of vision

It is generally assumed that multisensory integrative mechanisms take place in the brain, but the present findings show spinal effects, where visual signals were associated with decreases in the responses of the muscle afferents. We found that there was a decrease in the depth of modulation (δ) to repeated sinusoidal movements, when the participants viewed their foot moving. We verified that this change in muscle spindle sensitivity was not due to involuntary muscle activity, as the leg EMG activity recorded showed no significant differences across conditions. The effect of vision occurred independently of the attentional state of the participants, as manipulated via direct instructions to attend or not, where attention did not affect the δ. Similarly, there was no significant interaction between vision and attention. Therefore, we postulate that in the present experimental manipulation, attentional effects do not account for the changes in muscle spindle sensitivity in the different visual conditions. However, our manipulation of attention was explicit (i.e., we asked the participants to attend or not), which was in part constrained by the microneurography conditions where the participants are required to remain relaxed, and we were not able to confirm their attentional load. It may have been the case that participants may have simply disregarded the instruction to either attend or not attend; however, participants often reported the difficulty of the task, since the movements were actually all the same, which suggested that they really followed the instructions and executed the attentional task.

The absence of a change in muscle afferent activity with attention may appear contradictory with the results of previous studies, where it has been observed that a fusimotor-induced sensitization of muscle spindles occurs during proprioceptive attention tasks (Hospod et al., 2007; Ribot-Ciscar et al., 2009). However, the previous experiments were specifically designed to address the effect of attention, while here it was only a controlled parameter, and a difference between the difficulties of the present and previous tasks likely accounts for this. The present task was a simple comparison of movement amplitude at the end of the sinusoidal movements between attention conditions, which is far easier than the recognition of writing movements (Hospod et al., 2007) or classifying different movement amplitudes or velocities (Ribot-Ciscar et al., 2009).

We postulate that during the visual conditions, where the participant viewed their foot moving, the proprioceptive information, coupled with congruent visual signals, aids signal processing. In line with the Bayesian framework, a relative weighting of each visual and proprioceptive cue may account for the perceptual enhancement observed in our visuo-proprioceptive condition. The model predicts that if one sensory source becomes less reliable, the weight of the other one increases (Ernst and Banks, 2002). In the current study, when only proprioceptive information was available, the participant relied on one sensory source, where we found an increased sensitivity in firing of the muscle afferents. Conversely, when congruent visual information was present, the relative visual weight increased, while that of the proprioception decreased. Vision plays a dominant role in spatial tasks, as reported in studies using the mirror paradigm, where seeing the reflection of one’s moving arm in a mirror is sufficient to induce an illusion of a concomitant displacement of the other stationary, hidden arm (Guerraz et al. 2012). Furthermore, Chancel et al. (2016b) reported that illusions induced using the mirror paradigm can survive despite a marked visual impoverishment (obtained by covering between 0% and 100% of the mirror); the mirror illusion was significantly degraded only when the visual degradation was >84%, suggesting that even restricted visual information is sufficient to provide relevant kinesthetic cues. Future studies may be conducted to explore whether changing the reliability of the visual feedback by progressively degrading visual information results in an increase of muscle spindle sensitivity.

Previous microneurographic studies exploring the effect of vision on muscle proprioceptive information used external visual targets (Wessberg and Vallbo, 1995; Jones et al., 2001; Dimitriou, 2016), in contrast to our experiment where the participant viewed their own passive movement. Jones et al. (2001) described a decrease in muscle afferent firing rate during incongruent muscle afferent and visual feedback, which was interpreted as a strategy for resolving bisensory conflict. Conversely, an increase in muscle afferent firing was more recently observed during a similar visuomotor task specifically during an imposed adaptation phase, making the fusimotor control a means of adjusting the human proprioceptive system to motor learning (Dimitriou, 2016). Interestingly, the latter study also observed a decrease in muscle spindle dynamic sensitivity in the washout stage, when visual feedback was again congruent with muscle feedback. In line with Dimitriou (2016), we found that the fusimotor drive selectively decreased muscle spindle sensitivity when muscle afferent feedback was accompanied by congruent visual cues. Although at first glance they might seem disparate, taken together, these recent studies and our present one accounted for a fusimotor control of muscle spindle sensitivity independent of the concurrent muscle activity, which has long been debated (Vallbo et al., 1979). They all suggest that muscle spindle sensitivity may change according to its relevance to the context and, in particular, the presence or not of relevant visual cues.

The reweighting of proprioceptive information in the absence of visual signals can be related to the modulation observed in the primary somatosensory cortex depending on concomitant visual signals (Blakemore et al., 2005; Helbig et al., 2012). Using a design inspired by the Bayesian framework, Helbig et al. (2012) showed that during a task of shape identification, activation of the primary somatosensory cortex was modulated by the reliability of visual information within congruent visuo-tactile inputs. The less reliable the visual information, the more activity in the primary somatosensory cortex increased. In line with the modality appropriateness model (Welch and Warren, 1986) and the Bayesian framework (Ernst and Banks, 2002), one can assume that crossmodal processing is more likely to occur within the sensory pathway corresponding to the most accurate signal regarding the task, since this sensory signal is supposed to get a greater weight compared to the other less reliable signals.

Descending fusimotor influences from relevant visual cues may reduce the sensitivity of muscle afferents, reflecting a decrease in the proprioceptive contribution to encode the actual movement. Indeed, watching a video of one’s own hand in movement is sufficient to elicit an illusory movement of participant’s resting hand. By recording brain activity during this pure visually-induced kinesthetic illusion, Kaneko et al. (2015) found that the lateral premotor (PM) cortex and the supplementary motor area (SMA) were specifically activated together with the posterior parietal cortices and the insula. It is well known that the SMA and lateral PM are part of the motor system, with direct connections to M1, and descending output to the spinal cord (Dum and Strick, 1991; He et al., 1995; Picard and Strick, 1996, 2001; Maier, 2002). Further, the SMA and the lateral PM were not activated when participants viewed a video of someone else’s own hand. Only relevant kinesthetic visual cues may therefore influence proprioceptive sensitivity through descending motor commands that can modulate spinal fusimotor efference.

### Functional significance of the fusimotor modulation

One may consider that the observed fusimotor effect is small, as compared to in animals; however, it has been repeatedly observed in humans (Burg et al., 1975; Vallbo and Hulliger, 1981; Vallbo and Al-Falahe, 1990; Gandevia et al., 1994; Ribot-Ciscar et al., 2000, 2009; Jones et al., 2001; Hospod et al., 2007; Dimitriou, 2016; Ackerley et al., 2017) and has been considered as intriguing when compared to animal data, where muscle spindle firing rates are ten times higher than in humans, as are the fusimotor-induced changes (Matthews, 1981). Whatever its amount, the observed effect was sufficient to significantly alter activity of muscle afferents and hence may have a functional impact on the resulting perception.

Moreover, the fact that our two experiments have been done with two different populations of participants may at first appear as a limitation of the present study. However, it is worth noting that there is commonly a high variability in the firing of muscle afferents, depending on the nature and number of intrafusal muscle fibers that are included, the fusimotor innervation received by the intrafusal fibers, and the location of the muscle spindle inside the muscle, where a receptor near the ankle joint will be more affected by the movement than another located more proximally in the EDL or TA muscles. Therefore, one can consider that the variability introduced by the use of different participants does not overly influence the outcome, with respect to the intra-subject variability, due to the technical challenge of recordings made in the same subject.

Our finding that afferent proprioceptive signals from ankle could be modulated by visual cues may be important for controlling postural balance (Burke and Eklund, 1977; Massion, 1992; Kavounoudias et al., 2001). High ankle proprioceptive acuity has been observed to be predictive of sport performance level in elite athletes such as dancers (Han et al., 2015b) and in balance performance of the elderly (Goble et al., 2011). Similarly, better ankle proprioception is correlated with reduced ankle injuries (Han et al., 2015a), while after a complete loss of somatosensory afferents, deafferented patients present severe deficits in postural and motor tasks (Forget and Lamarre, 1995). The central processing of ankle proprioceptive information with other sensory information enables optimal integration for balance control. When a source of information is used for other purposes, for example, if vision is used to track a target in the environment, the CNS uses a reweighting strategy relying on the most reliable sources of information to optimize balance control. We presently show that a relative reweighting of visual signals may occur by a recalibration at more peripheral levels of ankle proprioceptive inputs, via a direct setting of muscle receptor sensitivity by the CNS.

The present results may have further clinical impact on sensorimotor rehabilitation. Different interventions are used to improve ankle proprioception and balance control, particularly after ankle injury. While passive interventions, such as taping or compressing, do not seem to particularly improve proprioception, active interventions with task-specific paradigms are efficient, suggesting central processing modifications (Han et al., 2015b). The present results suggest that removing visual information may optimize the intervention, by providing the brain with increased proprioceptive information that may favor a better recovery of balance control.

In conclusion, we show that muscle afferent sensitivity can be altered in a context-dependent way via descending influences. Specifically, we show that when proprioceptive signals from a foot movement are coupled with congruent visual information, a decrease in muscle afferent firing was found. This decrease in the bisensory condition may reflect a re-weighting of the two sensory cues in favor of the visual source. Our study shows that the mechanisms of sensory reweighting are not limited to higher-level neural control in the brain, but that there are also spinal effects of multisensory processing between visual signals and proprioceptive coding. This opens up the opportunity for the study of other multisensory effects below the level of the brain and impacts on our understanding of multisensory interactions throughout the CNS, which may also provide clinical therapeutic strategies for ameliorating visuo-sensorimotor disturbances.

## Synthesis

Reviewing Editor: Li Li, New York University Shanghai Decisions are customarily a result of the Reviewing Editor and the peer reviewers coming together and discussing their recommendations until a consensus is reached. When revisions are invited, a fact-based synthesis statement explaining their decision and outlining what is needed to prepare a revision will be listed below. The following reviewer(s) agreed to reveal their identity: Nestor Matthews.

The ms was reviewed by two experts in the field who reviewed the ms from two different angles. Both have concerns. Reviewer 1 raised some serious concerns about the experiment protocol and methodology which should be addressed before the ms can be considered for publication. I list the reviewers' comments below.

Reviewer 1

The article 'Seeing your foot move changes muscle proprioceptive feedback' is a very relevant study, as there has been a lot of discussions and a recent upsurge in activity in the field studying fusimotor systems in humans.

I enjoyed the introduction and based on that I presumed the authors were exploring the drive to the muscle spindle which alters its sensitivity, which can be measured but no so much from the afferent but from the γ-efferent. The afferent discharge is related to the activity of the spindle itself. Suggest a quick read from the Book 'The circuitry of the human spinal cord', chapter 3 on fusimotor mechanisms. Feel the authors have not quite defined the experimental premise well, although they do have the data to identify the answers to the questions raised, even if indirectly. I would like to rather have both studies conducted on the same subject (the qualitative evaluation of angle and the afferent firing information) during the same session, as that would allow for better correlations to be explored. I agree that the outcome change is likely to be due to a fusimotor drive if one assumes that the local inputs have all been stabilized within the small angles that their foot is moved within when the qualitative verification is implemented.

I have asked targeted questions and these are presented as comments in the pdf itself.

I have concerns with the protocol and would like to know if there was any evidence of fatigue? how were the 2 groups compared and normalized against what to ensure that they can be compared?

The methodology is not clearly written to enable someone to reproduce these outcomes. The choice of experimental design (randomized), with the choice of angles, is not at all clear.

Why did you classify the data based on multiple factors when your outcome was a binary outcome of increase or decrease compared to control, for the qualitative response of the subject to the angle they thought their foot had been moved through? Why not use something like Rasch analysis if you feel other variables are likely to influence the outcome? https://www.rasch.org/rmt/rmt213d.htm

Why did you record from an afferent if you wanted to record the fusimotor drive or did you want to record the change in frequency of spindle firing to assess if the presumed fusimotor drive was, in fact, modulating the output?

I found the argument tenuous at best that the modulation was due to the fusimotor input being modulated by vision, although it is likely it is not what the evidence suggests as these results are deduced and not observed.

The choice of angles between the 2 studies do not match which was another concern, neither did I know if the knee was restrained to ensure that the angle change in the foot was either allowing or disallowing a length change in TA/EDL muscles.

Think the study needs to be presented with a focus on the shift in the afferent response and evidence in support of a likely fusimotor drive change be better explained, which currently is not very clear.

Reviewer 2

The present study has the potential to contribute nicely to the intriguing possibility of afferent multisensory integration. Below I offer comments that I hope the authors find constructive.

The revised manuscript should greatly clarify the attention manipulation. Lines 170 - 173 state the following.

“To make sure that participants were attending, the participant was asked to judge whether it felt like the current sinusoidal movements were of larger movements than the previous ones, between attention conditions.”

Did participants make two responses on each trial (“One” vs “Two” to orally identify the movement with larger amplitude), and then a “Larger” vs “Smaller” response on the attentional question? Were correct vs incorrect responses possible on the attentional manipulation, and if so did participants receive accuracy feedback on their attention-related responses? Were the attention and no-attention trials randomly interleaved within a trial block, or were they separately blocked?

2. The present behavioral and physiological results showed no effects of attention. This could mean either that attention plays no role, or instead, that the manipulation was not effective. Can the authors provide evidence of a successful “manipulation check” to ensure that attention was in fact manipulated?

3, Figure caption 2A should explicitly state what IDL Ia represents as the y-axis label. Many readers will not understand that unit of measure.

4. The authors provide direct evidence for the visual cue significantly reducing muscle-afferent activity. However, the authors did not directly demonstrate that “the relative visual weight increased”, contrary to their claim in line 343. Consequently, the authors should describe that claim (about the relative visual weight) as speculative.

5. The authors speculation about cues being re-weighted according to reliability would be bolstered by citations showing greater spatial resolution in vision-alone than in proprioception-alone. One way to support that claim would be to cite some references on the well known hyper-acuities in vision ( https://en.wikipedia.org/wiki/Hyperacuity_(scientific_term) ).

## Author Response

Synthesis of Reviews:

Computational Neuroscience Model Code Accessibility Comments for Author (Required):

n/a

Significance Statement Comments for Author (Required):

n/a

Comments on the Visual Abstract for Author (Required):

n/a

Synthesis Statement for Author (Required):

The ms was reviewed by two experts in the field who reviewed the ms from two different angles. Both have concerns. Reviewer 1 raised some serious concerns about the experiment protocol and methodology which should be addressed before the ms can be considered for publication. I list the reviewers' comments below.

We wish to thank both reviewers for their relevant and very constructive comments. In particular, we realized that there was a lack of precision in the description of the methodology for readers who are not familiar with the microneurographic technique. We have taken into account all the issues they raised and have tried to address them as well as we could in the revised manuscript. Our point-by-point responses to the specific comments made are listed below.

Reviewer 1

The article 'Seeing your foot move changes muscle proprioceptive feedback' is a very relevant study, as there has been a lot of discussions and a recent upsurge in activity in the field studying fusimotor systems in humans.

I enjoyed the introduction and based on that I presumed the authors were exploring the drive to the muscle spindle which alters its sensitivity, which can be measured but no so much from the afferent but from the γ-efferent. The afferent discharge is related to the activity of the spindle itself. Suggest a quick read from the Book 'The circuitry of the human spinal cord', chapter 3 on fusimotor mechanisms. Feel the authors have not quite defined the experimental premise well, although they do have the data to identify the answers to the questions raised, even if indirectly. I would like to rather have both studies conducted on the same subject (the qualitative evaluation of angle and the afferent firing information) during the same session, as that would allow for better correlations to be explored. I agree that the outcome change is likely to be due to a fusimotor drive if one assumes that the local inputs have all been stabilized within the small angles that their foot is moved within when the qualitative verification is implemented.

I have asked targeted questions and these are presented as comments in the pdf itself.

Response: We would like to thank the reviewer who qualified our work as a relevant study. The reviewer subsequently reports different points that have been underlined throughout the paper in the form of various comments. We have copied and pasted each of these comments with the line of the PDF and responded precisely to all of them in a specific letter.

Here are the responses to the targeted questions.

1. I have concerns with the protocol and would like to know if there was any evidence of fatigue?

We realized from the reviewer's remark that we did not mention in the original manuscript that 3-minute breaks minimum were systematically applied every 20 pairs of movement comparisons during the psychophysical testing, and there were pauses in the microneurographic experiment. The experimenter regularly checked whether the subject needed to take an extra break at any time.

We have added detail about this on P.7 (Behavioral experiment) and P.8 (Microneurographic experiment).

To address the concern of the reviewer regarding an impact of the fatigue on the psychophysical results, we compared the judgments made by our participants during the first 7 versus the last 7 of the 15 repetitions in the vision and No vision conditions. As shown in the figures below, no systematic influence of the time was observed. The following two histograms represent the number of times (averaged across participants) the tested angle was perceived larger than the reference with and without vision.

During the microneurographic experiment, a time pause of 30 s duration was applied after each movement. Although no subject reported muscle fatigue, it cannot be completely excluded. However, muscle fatigue is known to impact spindle discharge during voluntary contraction where a 30% of maximum voluntary contraction is found to progressively decline spindle activity to half in about 1 minute (for 72% of the tested population of afferents, Macefield et al. 1991, J Physiol; 440: 497-512). Regarding muscle spindle sensitivity in response to passive imposed movements, to our knowledge there is no study which investigated the issue of the effect of fatigue.

2. how were the 2 groups compared and normalized against what to ensure that they can be compared?

Response: As indicated in the manuscript, the two experiments have been done with two different populations of participants and we agree with the reviewer that this may at first appear as a limitation of the study. However, performing all the tests in the same session was not possible because the present psychophysical tests lasted more than an hour and the microneurographic experiment lasted ~4 hours (due to the technical challenges, plus there is no guarantee to gain testable single unit muscle afferents). Hence, we would have exposed the participants to fatigue and loss of motivation with such delays.

In addition, it is worth noting that there is commonly a high variability in the firing properties of muscle afferents. This variability is related to the static and/or dynamic sensitivity of the muscle endings depending on the nature and number of intrafusal muscle fibers that are included, the fusimotor innervation received by the intrafusal fibers, and the location of the muscle spindle inside the muscle where a receptor near the ankle joint will be more affected by the movement than another located more proximally in the EDL or TA muscles of the leg. Therefore, one can consider that the variability introduced by the use of different participants does not overly influence the outcome, with respect to the intra-subject variability, due to the technical challenge of recordings made in the same subject.

This point has been added in the Discussion section P. 18 (bottom).

3. The methodology is not clearly written to enable someone to reproduce these outcomes. The choice of experimental design (randomized), with the choice of angles, is not at all clear.

Response: We have added further details on the methodology used and we more explicitly justified our experimental choices (see the point by point responses to the comments made in the pdf document and reported below). However, the interested reader who wants to know more about the microneurographic technique, is referred to other work (e.g. full details of our procedure precisely described in an already published book chapter (Bergenheim et al 1999 in Modern Techniques in Neurosciences Research), as per normal in such studies.

4. Why did you classify the data based on multiple factors when your outcome was a binary outcome of increase or decrease compared to control, for the qualitative response of the subject to the angle they thought their foot had been moved through? Why not use something like Rasch analysis if you feel other variables are likely to influence the outcome? https://www.rasch.org/rmt/rmt213d.htm

Response: In our opinion, our chosen model and the Rash model are not mutually exclusive, but address different aspects of the participants' perception.

The Rash model is about “how to measure the difference in ability between people engaged in a specific task” (Irwin, 2007). The corresponding model concerns the probability of success (while we compute the probability of a specific percept, e.g. “the test movement was bigger than the reference one”). In our opinion, this type of analysis would be relevant if we were interested in comparing our participants' performances instead of the change in their perception between different conditions (eyes closed/open) for the whole group.

Finally, we chose the approach classically used to estimate velocity discriminative thresholds of self-movements, as attested by our previous published studies (Chancel et al. 2016; Landelle et al. 2018), as well as many others in the domain (Wichman and Hill, 2001; Kingdom and Prins, 2011; Reuschel et al. 2009; Gori et al. 2011; Ernst & Banks 2002; Prsa et al 2012; Tagliabue and McIntyre, 2013...). It allowed us to compare our results directly with those from previous studies.

We have justified our choice in the revised manuscript (see P.8 & P.9)

5. Why did you record from an afferent if you wanted to record the fusimotor drive or did you want to record the change in frequency of spindle firing to assess if the presumed fusimotor drive was, in fact, modulating the output?

Response: In the present paper, we investigated whether the movement induced muscle afferent input was altered depending on the presence or absence of vision of the movement. In other words, we attempted to examine whether muscle spindle sensitivity changed in the presence of vision through the influence of the fusimotor drive. Therefore, we fully agree with the Reviewer that to address this issue, it would be ideal to record from gamma efferents directly. However, up to now, microneurographic recordings from such gamma efferents in humans are very rare. As far as we know, there exists only one paper based on the recording of gamma efferents in human and it has been published by our microneurographic team (Ribot et al. (1986), J Physiol London 375, 251-268). In this paper, it is stated that it took 5 years to record the unitary activity of only 6 gamma efferent fibers (see paragraph entitled “methodological consideration” in the discussion section of this paper) and the different reasons that could explain the difficulty in collecting these data were discussed. Today, even after 30 more years of microneurographic experience, the difficulty remains the same. Although we share the Reviewer's thoughts, we did not choose to set up experiments where the chance to approach such fibers is too small (~1/year!). Therefore, we have focused our studies for years on exploring this gamma drive in humans (for example depending on the behavioral or emotional contexts Hospod et al. 2007, Ribot-Ciscar et al. 2009, Ackerley et al. 2017), which are all based on extrapolations from the recordings of muscle afferents. Indeed, as alluded to by the Reviewer, we postulated that any change in frequency of spindle firing was likely to be due to a fusimotor drive modulation as soon as movements of the same angles were perfectly repeated (since passively imposed) and in the subject at rest.

We have now clarified this point, from the Introduction (P.3), so as to make it clear to the reader that our interpretations are made by the detour of afferent recordings and we have moderated our phrasing throughout the manuscript accordingly.

6. I found the argument tenuous at best that the modulation was due to the fusimotor input being modulated by vision, although it is likely it is not what the evidence suggests as these results are deduced and not observed.

Response: We agree with the reviewer that our conclusion is an interpretation. As mentioned in our original manuscript, we postulated that the modulation in muscle spindle responses was likely due to the fusimotor drive. However, we have reformulated our conclusions even more cautiously, as suggested by the reviewer.

7. The choice of angles between the 2 studies do not match which was another concern, neither did I know if the knee was restrained to ensure that the angle change in the foot was either allowing or disallowing a length change in TA/EDL muscles.

Response: We apologize for the absence of clarity regarding our experimental set-up.

In fact, since only one amplitude of movement was used in the microneurographic study, we choose the lowest amplitude (5{degree sign}) from the range of amplitudes previously tested in the present psychophysical tests, to minimize the risk of losing the unitary recording due to a displacement of the microelectrode that was more likely to occur due to the long lasting recording trials (30 cycles of ankle movement repeated). This point has been reported in the revised manuscript (see P.8).

It is also now specified that the knee was unrestrained but the foot was strapped on the pedal to make it driven by the displacement of the pedal with the ankle as the axis of rotation. (P.4)

8. Think the study needs to be presented with a focus on the shift in the afferent response and evidence in support of a likely fusimotor drive change be better explained, which currently is not very clear.

Response: Again, as recommended by the reviewer, we have clarified the purpose of the study, which was not based on a direct recording of fusimotor fibers, but on the recording of afferent muscle responses, whose modulation likely supports a change in the fusimotor drive.

Reviewer 2

The present study has the potential to contribute nicely to the intriguing possibility of afferent multisensory integration. Below I offer comments that I hope the authors find constructive.

The revised manuscript should greatly clarify the attention manipulation. Lines 170 - 173 state the following.

“To make sure that participants were attending, the participant was asked to judge whether it felt like the current sinusoidal movements were of larger movements than the previous ones, between attention conditions.”

Did participants make two responses on each trial (“One” vs “Two” to orally identify the movement with larger amplitude), and then a “Larger” vs “Smaller” response on the attentional question?

Were correct vs incorrect responses possible on the attentional manipulation, and if so did participants receive accuracy feedback on their attention-related responses?

Were the attention and no-attention trials randomly interleaved within a trial block, or were they separately blocked?

Response: We thank the reviewer and realized through his/her constructive remark that the description of the experimental procedure was lacking in clarity. In fact, it was always the same passive movement imposed to the participant, to compare the response of muscle afferents and investigate a change in firing properties of the afferent fibers depending on the experimental conditions independently of the muscle length. Therefore, participants could not receive any accuracy feedback on their attention-related responses.

In addition, the no-attention and attention trials were not randomized. To avoid any implicit attention task, the No-attention and Attention trials were blocked separately, and the No-attention block always preceded the Attention block.

These points have been added in the revised manuscript (P.8)

2. The present behavioral and physiological results showed no effects of attention. This could mean either that attention plays no role, or instead, that the manipulation was not effective. Can the authors provide evidence of a successful “manipulation check” to ensure that attention was in fact manipulated?

Response: The reviewer makes a very good point, which we have previously discussed. The present microneurographic experiment was set up to answer the question of whether looking at the moved foot or not impacted the movement-induced proprioceptive information. The comparison of vision and no-vision conditions on its own did not allow us to answer the question, since a change in attention between the two conditions might be responsible for a change in muscle proprioceptive information (see Hospod et al 2007, Ribot-Ciscar et al. 2009). To disentangle this difficulty, we added a condition where the “attention” was the only parameter under consideration. We agree with the Reviewer that we cannot be sure that participants were involved in the task consisting in comparing the movement amplitude in the two consecutive sequences. To investigate this issue, the most pertinent approach should have been to analyze the participants' responses, but analyzing these data was not adapted here since there was no correct or false response, with the movements being of same amplitude in both sequences. Electrodermal responses were recorded under this aim, but these parameters did not significantly change between “attention” and “no-attention” sequences, as often observed since these are more related to variation in emotion than in cognitive processes, therefore, we cannot provide more evidence of the participants' involvement in the task. However, participants often reported the difficulty of the task, since the movements were actually all the same, which suggested that they really followed the instructions and executed the attentional task.

3. Figure caption 2A should explicitly state what IDL Ia represents as the y-axis label. Many readers will not understand that unit of measure.

Response: We apologize for this omission. It is now specified in the figure caption that the recording is made from a primary muscle afferent (Ia) arising from extensor digitorum longus (EDL) muscle.

4. The authors provide direct evidence for the visual cue significantly reducing muscle-afferent activity. However, the authors did not directly demonstrate that “the relative visual weight increased”, contrary to their claim in line 343. Consequently, the authors should describe that claim (about the relative visual weight) as speculative.

Response: We fully agree with the reviewer that the present study does not directly demonstrate a relative visual weight increase and we have reformulated this speculative interpretation accordingly.

5. The authors speculation about cues being re-weighted according to reliability would be bolstered by citations showing greater spatial resolution in vision-alone than in proprioception-alone. One way to support that claim would be to cite some references on the well known hyper-acuities in vision ( https://en.wikipedia.org/wiki/Hyperacuity_(scientific_term) ).

Response: We thank the reviewer for this suggestion and have added some references to support the fact that vision predominates in spatial tasks. In particular, many studies have shown that it is possible to evoke illusory movement sensation based on visual inputs, despite the proprioceptive feedback that signals the absence of actual movement. This has been widely reported in studies using the mirror paradigm showing that when participants look at the reflection of their moving left arm in a mirror, they feel an illusion of a concomitant displacement of their stationary, hidden right arm (Guerraz et al. 2012). Recently, we found that illusions induced using the mirror paradigm can survive despite a marked visual impoverishment (obtained by covering between 0 and 100% of the mirror in 16% steps): the mirror illusion was significantly degraded when the visual degradation reaches a minimum of 84%, suggesting that a limited amount of visual information is enough to provide relevant kinesthetic cues (Chancel et al. Exp Brain Res 2016).

Responses to the pdf comments made by Reviewer 1

P0 l11 : do you mean the firing properties of the afferent changed? or the response as in the evoked response?

As alluded to the reviewer, it is the fact that for the same passive movement, the response of muscle afferents increased when the proprioceptive channel was the only source of information, as compared with when visual cues were added, which suggested a change in firing properties of the afferent fibers.

This point has been specified in the revised manuscript P.1

P1 l 25 too vague and not clear how and why this would be expected or be of use for sensorimotor rehabilitation.

The sentence has been replaced by the following: 'These results impact sensorimotor rehabilitation, where clinical strategies using exercises without visual feedback may promote proprioceptive training'. (P.1)

73 : am not sure if I am reading this right, but when one talks about the proprioceptive inputs from a muscle it is the afferent drive which is the state of the spindle that is being transferred to the central nervous system, unlike what seems to discussed here which is the gamma drive. the gamma system is useful and defines the sensitivity of the spindles but does not work alone.

We agree that what is highlighted here is the involvement of the gamma drive in motor learning processes. The sentence has been changed accordingly (P.3)

79: the assumption here appears to be that the muscle in passive state when moved by another device or person, is not going to undergo change in length, which would affect the spindle output or provide feedback about the state of the muscle. Passive movement which induce isometric movement of the muscle too are known to lead to minor lengthening of the muscles involved. It too can influence the output.

In fact, in our experiment, we imposed passive movements which actually changed muscle length and this activated muscle spindle endings. Furthermore, because it was a passive movement, there was no concomitant activation of the skeletomotor neurons, i.e. an alpha-gamma coactivation, which likely modifes muscle spindle properties (see explanation P.4).

84: how was this defined?

Only muscle proprioception was obtained when participants kept their eyes closed. This precision has been added in the manuscript (P.4).

90: how did you randomise if the 2 groups as listed later, did not actually take part in both studies but only any one of these. So participants in first did not take part in 2nd and vice-versa? if so how is the afferent and behaviour data matching up?

Please see response 2 on this point in the letter of responses to reviewers

102: why 120-130 and not 0? is this the knee extension or flexion angle?

We apologize for the lack of clarity - the subjects were in a seated position with their knee joint at a flexion angle of ~120-130{degree sign}. (P.5)

106: Suspect you mean pairs of electrodes were placed on each muscle or do you mean 2 per muscle? Also, what was their make and kind? how did you decide on the location for recording the EMGs from the muscles and the site where you recorded the neurograms from? What kind of landmarks were used to identify these locations?

A pair of surface electrodes (Ag-AgCl, inter-electrode distance 2 cm) was placed over the tibialis anterior (TA) and another pair on gastrocnemius soleus (GS) muscle bellies during the behavioral experiment. The location of each pair of electrode was defined by asking the participant to isometrically contract the muscle under consideration and palpation of the muscle belly.

These clarifications have been added to the revised manuscript (P.5)

121: differentially I suspect not referentially.

We agree with the reviewer that this is a better term and have changed it in the manuscript accordingly (P.5).

124: why was a 300-3000Hz the bandwidth of choice? Are you not loosing most of the slow events while 3000hz being at the low end of firing frequency of most axons, especially if they are like the muscle spindle secondaries which fire at high frequencies but for very short intervals. The SNR is not the best argument for choice of filter but the fact that you possibly used it to match the nyquist band width of the EMG and the afferent signal, although your sampling frequency was too high for something being captured at 3000hz.

We can see the reviewer's concern, yet we find the chosen bandwidth to be best suited to discriminate the waveform signal (note, this is not applied to the firing frequency) muscle afferents clearly. We have used the filter settings for a long time (nearly 40 years in the present microneurography lab, e.g. Roll & Vedel, 1982, Exp Brain Res; Ribot et al., 1986, J Physiol), thus it is purely based on optimizing our signal-to-noise ratio and not on other factors in the current recording (e.g. our EMG). This range is also similar to that of other microneurography labs (e.g. Löken et al., 2009, Nat Neuro; Ackerley et al., 2014, J Neurosci) where 200-4000 Hz is typical, and the original microneurography papers suggest a high pass filter of 300 Hz (Vallbo & Hagbarth, 1968, Exp Neurol), with a variable low-pass. To be clear, the bandpass filter is only used on our real-time recorded waveform, not during off-line processing, e.g. of the firing rate that is extracted afterwards. The microneurographic technique consists in making axonal recordings and the typical spike duration is 1 ms. Therefore with a 300-3000 Hz bandwidth, the waveform of each spike is clearly defined meaning that there is no risk for its amplitude being cut because of a low sampling rate. We regularly record from large amplitude spikes, which are preferable in our recordings for the clarity of the data (e.g. see Figure 2). Regarding the low frequency, there is no need in the nerve signal to record slow events; on the contrary these could be events that disrupt the signal such as the 50 Hz and its harmonics decreasing the signal-to-noise ratio.

129: What are these?

'Conditions' referred to: vision, no-vision, attention, no-attention conditions

It has been added in the manuscript (P.6)

130: where were these recordings made and how was the orgin or the source identified?

The origin of the spindle afferents was determined by pressing the tendon of each particular muscle and the one triggering a nerve activity is considered as the endowed muscle. (P.5)

137: was the ankle and knee fixed in place? how did you ensure EDL was free of any activity if the ankle was not stabilised, above it states the leg was rested in a fixed position on a platform. how was the stability of this defined and maintained.

As mentioned before, it is now specified that the knee was unrestrained but the foot was strapped on the pedal to make it driven by the displacement of the pedal with the ankle as the axis of rotation. (P.4)

140: angle of what to what? assuming it is the angle at the ankle of the foot to the shin bone?

Yes, the reference movement corresponded to an amplitude angle of 6.4{degree sign} between the foot and the shin bone. (P.6)

140: repeated how often, noticed it is mentioned later but having it mentioned here would make it easier to read. Was the order in which they were tested randomised, so the sequence of tests, vision versus no vision could not be predicted by the participants?

Yes, vision and no-vision trials were randomized to prevent any anticipation and prediction from the participants. (P.6)

146: how and why was this range chosen? do you know if the difference in angles is sufficient enough to be detected as being different from the refernce angle used? any pilot or previously published study that supports these angles used?

As guessed by the reviewer, these angles were chosen on the basis of a previously defined pilot study (performed on 4 participants not included in the main experiment), in order to identify angle amplitudes that make discrimination against the 6.4{degree sign} reference very difficult (6 and 6.8{degree sign}) or rather easy (5.1 and 7.6{degree sign}) or of intermediate difficulty (5.6 and 7.2{degree sign}).

This point has been added in the revised manuscript (P.6)

150: how often and for how long? as fatigue of the ankle would introduce a feedback that would be different from that under normal conditions. this would be peripheral fatigue of the muscle, especially the EDL if you are modifying the ankle position. How long did the robot maintain the position?

Please see the main response 1 on this point in the letter of responses to reviewers

154: this does not seem similar to the range presented earlier for the passive task, as there the range is from 5.1 to 7.6, which is a meagre 2.5 and not 5 as done here?

Please see main response 7 on this point in the letter of responses to reviewers

also, if at a frequency of 1/s that is a fairly high frequency, did you evaluate if the EMG amplitude in EDL was affected significantly?

As mentioned in the manuscript, no significant changes in EMG amplitude were detected in any muscle recorded (Table 2)

157: not sure I understand why this is easier if the length is longer? afferent discharge is related to muscle lengthening, which is not an isometric condition which is what the authors described in introduction when mentioning Edin and Valbo 1990.

We have further developed the justification of the longer movement protocol we chose. In fact, it was crucial to record muscle afferent firing in the absence of muscle activity so that:

1) participants remained relaxed over a longer period of time than if we repeated movements every 2 s with interruption;

2) if a contraction did occur, despite the instruction to relax, the contaminated movement cycles could be removed without compromising the ability to extract an averaged response; and

3) because of a well-known dynamic response in sensory discharge at the onset of the movement (see red arrow on the figure below, see Edin and Vallbo 1990), the first cycle was systematically removed from the analysis; if we had chosen pairs of movements, it would have required too many repetitions of trials, due to a larger number of removed responses (which would have increased the risk of losing the unitary recording).

158: how was it decided that it was inadvertent? why is this not affecting the total average if all you have are 15 traces per subject, a statistically relevant outlier measure should have been employed.

During the microneurography recordings, we wanted the participant to be relaxed, hence show no fluctuations in the EMG. We did not particularly need to use special measures to identify EMG activity, as the EMG trace was either at a continuous and steady level (flat) or there was obvious EMG. We found very little EMG anyway, as the participants were calm throughout, which was required of them. We have re-written the part in the manuscript to be more accurate and moved it to the microneurography analysis section:

'The first cycle of the movement was not analyzed because of a dynamic response from the onset of the movement. Occasionally, some EMG activity (i.e. fluctuations in the steady EMG baseline) was found, despite the instruction for the participant to relax. When this occurred, the contaminated movement cycle was removed (cf. Ackerley et al., 2017). This occurred in only 5/64 runs (16 units x 4 conditions) and for each case, at least 85% of cycles were included.'(P.8)

160: if using a robot surely you got an effect of inertia both at the start and and the end of the series of tasks.

In fact, the actual movements of the robot recorded during the 30 cycle repetitions did not reveal any difference between all the cycles from start to end as shown in the figure below, i.e. see the first trace on the top of the figure which is the actual displacement of the pedal and not the command signal of the pedal. In addition, since the first cycle was systematically removed from the analysis, any inertia phenomenon will not have impacted the results.

161: 80% of 15 = so at least 12 per subject per angle?

In fact, 30 and not 15 foot displacements were imposed for each condition which resulted in at least 24 cycles per subject and per condition.

163: how was this evaluated and measured? was microneurography used to define activity or meagrely based on what they felt? if after the task it is very unlikely to be direct measure of the sensitivity of the spindles but that of the altered output.

We have removed this point in accordance with the reviewer's remark.

173: too qualitative

The sentence has been corrected.

182: apologies, if i sound stupid but why do you need a fancy gaussian with multiple assumed parameters when the outcome at each angle is one of 3 possibilities, same, higher or lower. anything else is what you are assuming based on the other paramters introduced to the argument. quite simply within the 2.5 degree range did the subjects identify the sinusoidal amplitude to be the same, higher or lower is the first pass, the gaussian can be interesting if the predicted amplitude matches at some angles but not others, otherwise this is a measure that is experimenter designed or in other words simulated and not obtained experimentally.

We thank the reviewer for pointing out that our choice of a psychophysical discrimination task needs to be further justified. In our design, participants had to choose between higher or lower (they could not answer “same”). We used a constant stimulus method meaning that seven intensities of movement were paired with the reference movement, and presented to participants 30 times in a random order, avoiding anticipation and predictability effects. The psychophysical method used to fit the raw data to a Gaussian curve has been widely used in many studies investigating human perception and proved efficient to capture individual specificity and biases in various perceptive tasks including regarding the self-movement perception (Wichman and Hill, 2001; Kingdom and Prins, 2011; Reuschel et al. 2009; Chancel et al. 2016). In this fitting procedure (performed using the Psignifit toolbox), bootstrap analysis are performed and the goodness-of-fit of the chosen model (i.e. the Gaussian function) is checked. As a result, the statistical power of the two parameters obtained to describe each participant's perception, mean and variance, are reinforced, which in our opinion leads to a more reliable comparison between the different conditions both within and between participants.

See more details added in the revised manuscript (P.9-10)

196: seriously object to the use of t-test for this as the parametric nature of the data was not established, and also the measure is a derived value so not suited for comparison of effect unless we know that each individual guessed it similarly each time during the 15 trials in each condition, at each angle.

We agree with the reviewer that the discriminative thresholds can be assimilated as positively skewed continuous variables modeled by a Gamma distribution. Thus, we have used a non-parametric generalized linear model (GzLM) for repeated measures to compare these variables between the two visual conditions. GzLM analysis showed that sigma values were significantly higher in the Vision condition compared to No vision (slope = 0.2422, t = 3.306, p &lt; 0.0001).

Changes in the revised manuscript have been added in the Method (P.10) and Results (P.12).

201: unless my calculations are wrong, is this not 200Hz? which is below lowest cutoff frequency used for data collection. why was this bin size chosen?

In microneurographic experiments, the data collected is the nerve signal as a waveform (see figure 2, the second trace from the top). From this waveform, we extracted the time occurrence of each spike (using the Spike2 program, by means of template recognition), which was marked. From this series of time-stamped occurrences (spikes), we calculated the instantaneous frequency, which was interpolated as an instantaneous frequency curve with a 0.005 s (1/0.005 = 200 Hz) bin size that gives an accurate representation of the unitary firing frequency (see figure 2, the first trace from the top). Even when bursts of muscle afferent activity are present, they are rarely higher than 50 Hz in humans under such conditions and never higher than 200 Hz.
